# Peroxisome biogenesis in mammalian cells

**DOI:** 10.3389/fphys.2014.00307

**Published:** 2014-08-15

**Authors:** Yukio Fujiki, Kanji Okumoto, Satoru Mukai, Masanori Honsho, Shigehiko Tamura

**Affiliations:** Department of Biology, Faculty of Sciences, Kyushu University Graduate SchoolFukuoka, Japan

**Keywords:** CHO cell mutants, genetic phenotype-complementation, import machinery, membrane assembly, pathogenic genes, peroxins, peroxisome targeting signals, Zellweger syndrome

## Abstract

To investigate peroxisome assembly and human peroxisome biogenesis disorders (PBDs) such as Zellweger syndrome, thirteen different complementation groups (CGs) of Chinese hamster ovary (CHO) cell mutants defective in peroxisome biogenesis have been isolated and established as a model research system. Successful gene-cloning studies by a forward genetic approach utilized a rapid functional complementation assay of CHO cell mutants led to isolation of human peroxin (*PEX*) genes. Search for pathogenic genes responsible for PBDs of all 14 CGs is now completed together with the homology search by screening the human expressed sequence tag database using yeast *PEX* genes. Peroxins are divided into three groups: (1) peroxins including Pex3p, Pex16p, and Pex19p, are responsible for peroxisome membrane biogenesis via classes I and II pathways; (2) peroxins that function in matrix protein import; (3) those such as three forms of Pex11p, Pex11pα, Pex11pβ, and Pex11pγ, are involved in peroxisome proliferation where DLP1, Mff, and Fis1 coordinately function. In membrane assembly, Pex19p forms complexes in the cytosol with newly synthesized PMPs including Pex16p and transports them to the receptor Pex3p, whereby peroxisomal membrane is formed (Class I pathway). Pex19p likewise forms a complex with newly made Pex3p and translocates it to the Pex3p receptor, Pex16p (Class II pathway). In matrix protein import, newly synthesized proteins harboring peroxisome targeting signal type 1 or 2 are recognized by Pex5p or Pex7p in the cytoplasm and are imported to peroxisomes via translocation machinery. In regard to peroxisome-cytoplasmic shuttling of Pex5p, Pex5p initially targets to an 800-kDa docking complex consisting of Pex14p and Pex13p and then translocates to a 500-kDa RING translocation complex. At the terminal step, Pex1p and Pex6p of the AAA family mediate the export of Pex5p, where Cys-ubiquitination of Pex5p is essential for the Pex5p exit.

## Introduction

Molecular mechanisms of peroxisome biogenesis, including peroxisomal import of newly synthesized matrix and membrane proteins, have been one of the major foci in the peroxisome research. Studies at the molecular level on both peroxisome assembly and peroxisome biogenesis disorders (PBDs) rapidly progressed in the last three decades. Studies on cloning of genes, particularly including those of a very low-level expression, have benefited from so-called functional cloning of genes, mostly cDNAs in mammalian cases, by phenotype complementation assay using cell mutants defective of biological pathways. The identification and characterization of numerous essential genes, termed *PEX*s encoding peroxisome biogenesis factors termed peroxins, by means of the genetic phenotype-complementation of peroxisome assembly-defective cell mutants, named *pex* mutants impaired in *PEX* genes. Such mutants from Chinese hamster ovary (CHO) cells (Table 1; see below) (Fujiki, 1997, 2000), several yeast species including *Saccharomyces cerevisiae* (Erdmann et al., 1989), *Pichia pastoris* (Gould et al., 1992; Liu et al., 1992), *Hansenula polymorpha* (Cregg et al., 1990), and *Yarrowia lipolytica* (Nuttley et al., 1993) (also see reviews Van Der Klei and Veenhuis, 1996; Kunau, 1998; Tabak et al., 1999; Subramani et al., 2000; Titorenko and Rachubinski, 2001; Lazarow, 2003), and plant *Arabidopsis thaliana* (Hayashi and Nishimura, 2006) have made invaluable contributions to the investigations of peroxisome biogenesis and protein trafficking in eukaryotes (Schatz and Dobberstein, 1996; Wickner and Schekman, 2005). We herein summarize mammalian model cell systems in studying biogenesis, physiology, and human disorders of peroxisomes.

**Table 1:** **Complementation groups (CGs) and *PEX* genes of peroxisome deficiencies**.

**Gene**	**CG**		**Phenotype**	**CHO mutants**	**Peroxisome ghosts**	**Peroxin**	
	**US/EU**	**Japan**				**(kDa)**	**Characteristics**
*PEX1*	1	E	ZS, NALD^*^, IRD^*^	Z24, ZP107	+	143	AAA family
*PEX2*	10	F	ZS, IRD^*^	Z65	+	35	PMP, RING
*PEX3*	12	G	ZS	ZPG208	−	42	PMP, PMP-DP
*PEX5*	2		ZS, NALD	ZP105^*^, ZP139	+	68	PTS1 receptor, TPR family
*PEX6*	4(6)	C	ZS, NALD^*^	ZP92	+	104	AAA family
*PEX7*	11	R	RCDP	ZPG207	+	36	PTS2 receptor, WD motif
*PEX10*	7(5)	B	ZS, NALD		+	37	PMP, RING
*PEX11β*	16		ZS		+	28	PMP
*PEX12*	3		ZS, NALD, IRD	ZP109	+	40	PMP, RING
*PEX13*	13	H	ZS, NALD^*^	ZP128	+	44	PMP, PTS1-DP, SH3
*PEX14*	15	K	ZS	ZP110	+	41	PMP, PTS1-DP, PTS2-DP
*PEX16*	9	D	ZS		−	39	PMP, PMP-DP
*PEX19*	14	J	ZS	ZP119	−	33	CAAX motif, PMP receptor
*PEX26*	8	A	ZS, NALD^*^, IRD^*^	ZP124, ZP167	+	34	PMP, Pex1p-Pex6p recruiter
				ZP114	+		

* ,Temperature-sensitive phenotype.

ZS, Zellweger syndrome; IRD, infantile Refsum disease; NALD, neonatal adrenoleukodystrophy; RCDP, rhizomelic chondrodysplasia punctata; DP, docking protein; PMP, peroxisome membrane protein; TPR, tetratricopeptide repeat.

## Genetic approaches to studying mammalian peroxisome biogenesis

Basically two mutually complementary approaches have been taken for isolation of *PEX* genes encoding peroxins, i.e., the genetic phenotype-complementation of peroxisome biogenesis-defective mutants of mammalian somatic cells such as CHO cells and a combination of the human ortholog isolation by homology search on the human expressed sequence tag (EST) database using yeast *PEX* genes and cells derived from the patients with PBDs of 14 different genotypes, i.e., complementation groups (CGs) (Table 1; see below) (Fujiki, 1997, 2000, 2003; Gould and Valle, 2000; Weller et al., 2003).

### Mammalian cell mutants deficient of peroxisome

**Figure 1:**
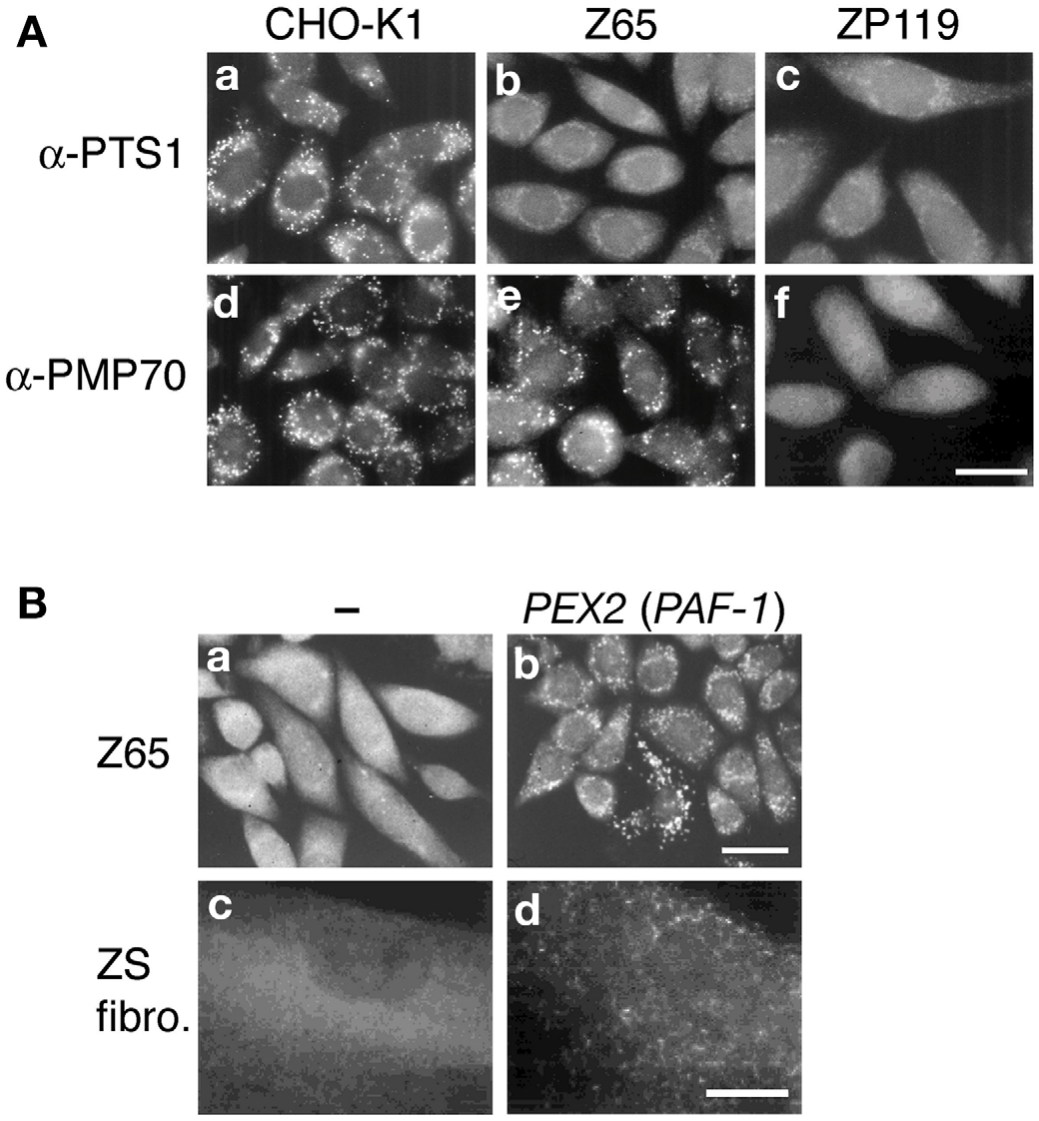
**Morphology of peroxisomes in CHO cell mutants defective in peroxisome biogenesis and cloning pathogenic genes of PBDs. (A)** Cells are stained with antibodies to PTS1 **(a–c)** and PMP70 **(d–f)**. Cells are as indicated at the top. Scale bar, 20 μm. In contrast to the wild-type CHO-K1 cells, PTS1 proteins are discernible in the cytosol in *pex2* Z65 and *pex19* ZP119. Z65 contains PMP70-positive peroxisomal remnants, whilst ZP119 is absent from such peroxisome ghosts, indicative of the defect of membrane protein import. **(B)** Cloning of pathogenic gene of PBD. Peroxisome-restoring *PEX* gene were isolated by functional complementation assay using CHO mutant. Restoration of peroxisomes in Z65 **(a)** by transfection of rat liver cDNA library **(b)**. Transformed cells positive in catalase import contained *PAF-1* (*PEX2*). In fibroblasts from a patient with ZS of CG10 **(c)**, expression of *PAF-1* restored the impaired import of catalase **(d)**. Scale bar, 20 μm **(a,b)**; 30 μm **(c,d)**.

Genetic heterogeneity consisting of 14 CGs were identified in PBDs by cell-fusion CG analysis using fibroblast cell lines derived from PBD patients (Fujiki, 2000; Ghaedi et al., 2000a; Gould and Valle, 2000; Matsumoto et al., 2001), where CGs 4 and 7 were revealed to be the same CGs as CGs 6 and 5, respectively (Table 1). A new CG, CG15, of ZS was also identified (Shimozawa et al., 2004), hence indicative of totally 13 genotypes of PBDs. The primary defect for PBDs was revealed to be the impaired biogenesis of peroxisomes (Fujiki, 2000; Gould and Valle, 2000). With respect to somatic animal cell mutants, 12 CGs of peroxisome-deficient CHO cell mutants were isolated, including a mutant ZP114 of a CG distinct from human CGs (Figure 1; Table1). A PBD patient of the 14th CG, CG16, was recently identified with pathogenic gene *PEX11β* (Ebberink et al., 2012). Together, genetic heterogeneity comprising 15 CGs are currently identified in mammals including humans and CHO cells.

### Peroxisome biogenesis genes

#### Genetic phenotype-complementation screening

*PEX*s were isolated by genetic phenotype complementation of peroxisome biogenesis-deficient mutants of mammalian somatic cells including CHO cells (Figure 1A) and of several yeast species including *S. cerevisiae*, *P. pastoris*, *H. polymorpha*, and *Y. lipolytica* (Distel et al., 1996; Subramani et al., 2000; Fujiki et al., 2006b). Two mutually distinct but complementary approaches have been taken to identify and clone mammalian *PEX* genes.

**Figure 2:**
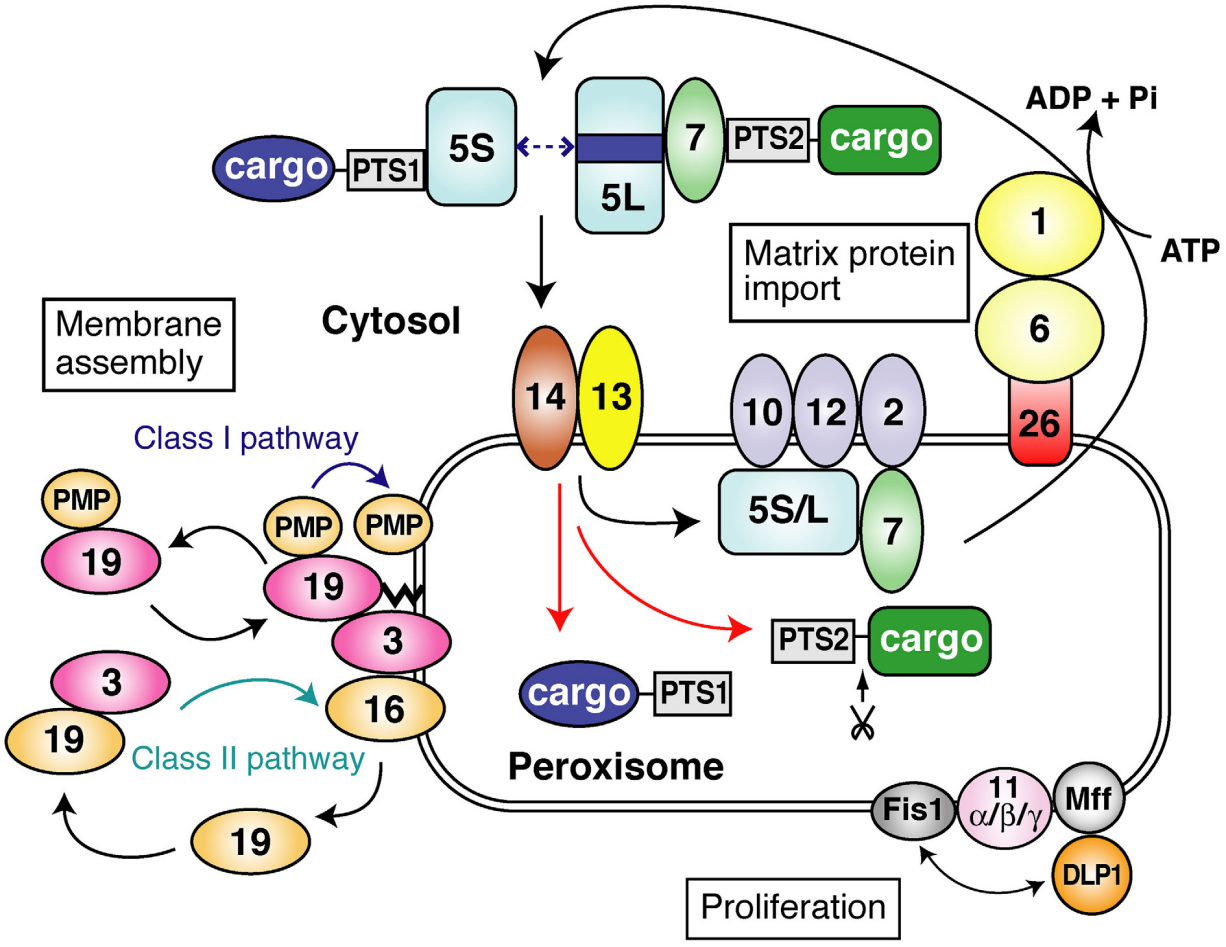
**A schematic view of peroxisome biogenesis in mammalian cells**. The subcellular localization and molecular characteristics of peroxins are shown. Peroxins are classified into three groups: (1) peroxins that are required for matrix protein import; (2) those including Pex3p, Pex16p and Pex19p, responsible for peroxisome membrane assembly via classes I and II pathways (see in this figure); (3) those such as three forms of Pex11p, Pex11pα, Pex11pβ, and Pex11pγ, apparently involved in peroxisome proliferation where DLP1, Mff, and Fis1 coordinately function. PTS1 and PTS2 proteins are recognized by Pex5p and Pex7p, respectively, in the cytoplasm. Two isoforms, Pex5pS and Pex5pL, of Pex5p are identified in mammals. PTS1 proteins are transported by homo- and hetero-oligomers of Pex5pS and Pex5pL to peroxisomes, where Pex14p functions as a convergent, initial docking site of the “protein import machinery” translocon. Pex5pL directly interacts with the PTS2 receptor, Pex7p, carrying its cargo PTS2 protein in the cytosol and translocates the Pex7p–PTS2 protein complex to Pex14p. PTS1 and PTS2 proteins are then released at the inner surface and/or inside of peroxisomes, downstream Pex14p and upstream Pex13p. Pex5p and Pex7p subsequently translocate to other translocon components, named translocation complex comprising the RING peroxins, Pex2p, Pex10p, and Pex12p. Both Pex5p and Pex7p finally shuttle back to the cytosol. In regard to peroxisome-cytoplasmic shuttling of Pex5p, Pex5p initially targets to an 800-kDa docking complex containing Pex14p and then translocates to a 500-kDa translocation complex comprising RING peroxins. At the terminal step of the protein import reaction, Pex1p and Pex6p of the AAA family catalyze the export of Pex5p, where Cys-ubiquitination of Pex5p is prerequisite to the Pex5p exit.

A direct cloning approach has been taken by means of genetic complementation with peroxin cDNA essential for assembly of peroxisomes in CHO cells. Establishment of an effective method, termed P12 (12-(1′-pyrene)dodecanoic acid)/ultraviolet selection method, made it feasible to isolate revertant (transfectant) cells showing a morphologically and biochemically normal peroxisome-phenotype, whereby *PEX2* (formerly *PAF-1*) encoding the 35-kDa membrane peroxin Pex2p with RING zinc-finger motif was cloned for the first time (Tsukamoto et al., 1991) (Figure 1B). Expression of *PEX2* (called *Zellweger gene*) in fibroblasts from a ZS patient of CG10 (F) complemented the impaired peroxisome biogenesis (Shimozawa et al., 1992) (Figure 1B). Dysfunction of *PEX2* caused by a homozygous nonsense point mutation at R119ter was shown for the first time to be responsible for ZS, a prototype of the PBDs (Shimozawa et al., 1992). A more practical approach, i.e., a transient expression assay, was also developed for further isolation of *PEX* cDNAs including nine others, *PEX1*, *PEX3*, *PEX5*, *PEX6*, *PEX12*, *PEX13*, *PEX14*, *PEX19*, and *PEX26* (Fujiki, 2003; Fujiki et al., 2006b) (Figure 2). These *PEX*s were shown to be the pathogenic genes involved in PBDs of nine CGs (Weller et al., 2003; Fujiki et al., 2006b; Fujiki, 2011) (Table 1).

#### Expressed sequence tag homology search

As an alternative method, the homology search by screening the human EST database using yeast *PEX* genes successfully led to isolation of human ortholog genes responsible for PBDs: *PEX1*, *PEX3*, *PEX5*, *PEX6*, *PEX7*, *PEX10*, *PEX12*, *PEX13*, and *PEX16* (Weller et al., 2003; Fujiki et al., 2006b).

All of pathogenic genes responsible for PBDs of currently identified 13 CGs have been successfully cloned within about 10 years after the first isolation of the ZS gene, *PEX2*, by such extensive search using the mutually complementary methods.

## Biogenesis of peroxisomes

### Membrane biogenesis

Three mammalian peroxins, Pex3p, Pex16p, and Pex19p, were isolated by the functional phenotype-complementation assay on CHO cell mutants (Matsuzono et al., 1999; Ghaedi et al., 2000b) and the EST database search using yeast *PEX* genes (Kammerer et al., 1997, 1998; Honsho et al., 1998; South and Gould, 1999) and were shown to be exclusively required for membrane assembly of peroxisomes. Mechanistic insights on membrane biogenesis are addressed here.

#### Peroxins essential for membrane assembly of peroxisomes

**Figure 3:**
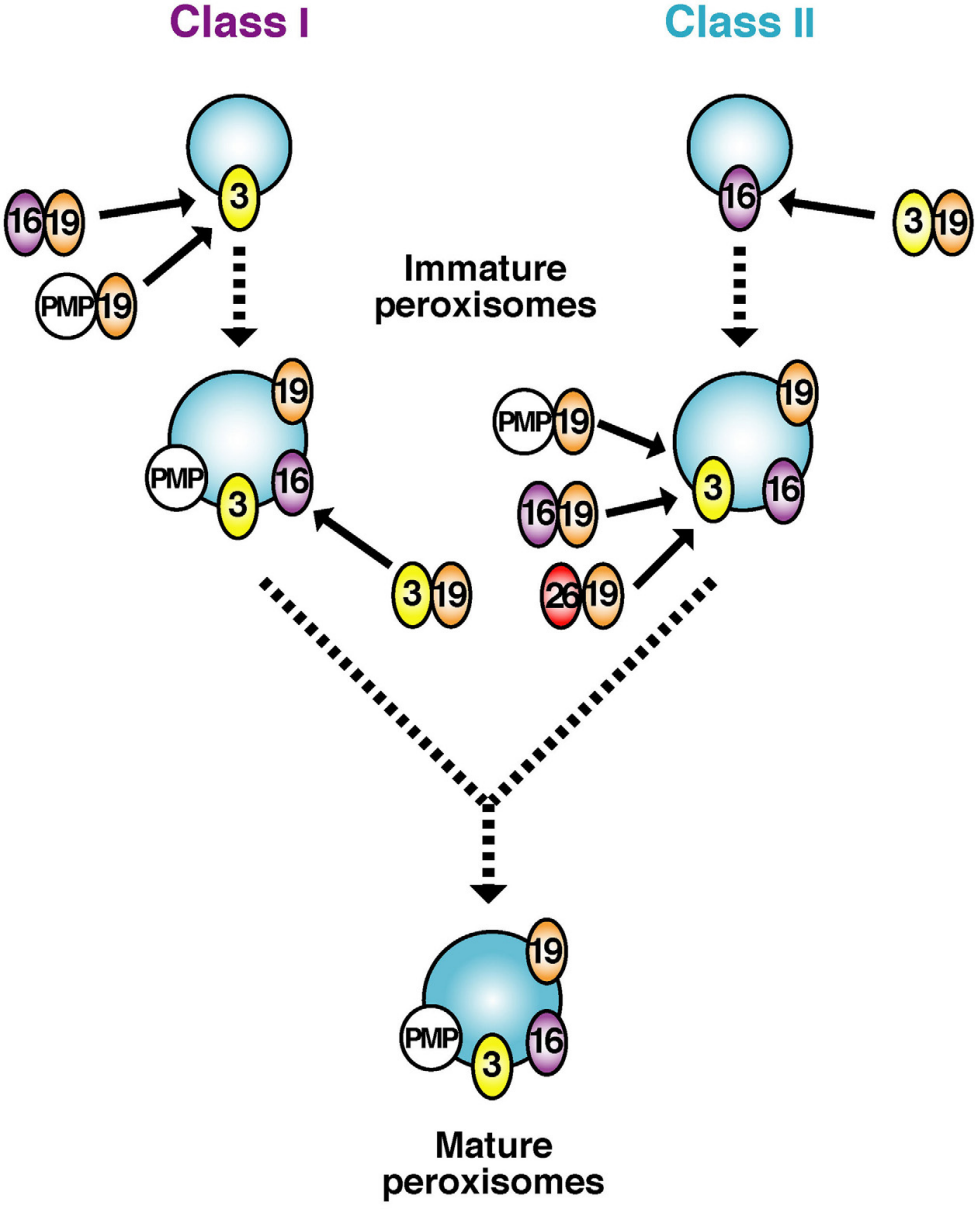
**A model for early stages of peroxisomal membrane biogenesis involving mutually dependent targeting of Pex3p and Pex16p, named classes I and II pathways**. The initial membranes harboring Pex3p or Pex16p culminate in indistinguishable, matured peroxisomes. Pex19p forms complexes in the cytosol with newly synthesized PMPs including Pex16p and C-tailed anchored membrane proteins such as Pex26p and transports them to the membrane protein receptor Pex3p, whereby peroxisome membrane is assembled (Class I pathway). With respect to biogenesis of Pex3p, Pex19p likewise forms a complex with newly synthesized Pex3p and translocates it to the Pex3p receptor, Pex16p (Class II pathway). Of note, peroxisomes are assembled no matter which pathway initially proceeds.

Of 13 peroxins of which mutations are responsible for PBDs, Pex3p, Pex16p, and Pex19p were identified as essential factors for PMP assembly in several species including humans (Baerends et al., 1996; Götte et al., 1998; Honsho et al., 1998; Matsuzono et al., 1999; South and Gould, 1999; Ghaedi et al., 2000a; Hettema et al., 2000; Sacksteder et al., 2000; South et al., 2000; Otzen et al., 2004) (Figure 1). Pex19p is a predominantly cytoplasmic protein that shows a broad PMP-binding specificity; Pex3p serves as the membrane-anchoring site for Pex19p-PMP complexes (Class I pathway); and Pex16p—a protein absent in most yeasts (Eitzen et al., 1997; South and Gould, 1999) functions as the receptor for Pex19p complexes with newly synthesized Pex3p (Matsuzaki and Fujiki, 2008) (Class II pathway) (Figures 2, 3). The function of Pex16p is not conserved between different species. In addition, under debate remains whether Pex19p has a chaperone-like role in the cytosol or at the peroxisome membrane and/or functions as a cycling import receptor for newly synthesized PMPs (Fujiki et al., 2006a).

#### Gene defects of peroxins required for both membrane biogenesis and matrix protein import

Impairment of Pex3p, Pex16p, and Pex19p, causes the most severe PBD, ZS, of three CGs, CG12 (G), CG9 (D), and CG14 (J), respectively (Weller et al., 2003; Fujiki et al., 2006b, 2012; Fujiki, 2011) (Table 1).

***Pex19p***. *PEX19* encodes 33-kDa farnesylated protein harboring farnesylation CAAX box motif localized mostly in the cytosol and only partly anchored to peroxisomal membranes (Matsuzono et al., 1999). *PEX19* expression complemented impaired peroxisome assembly in fibroblasts from a patient with CG14 (J) PBD. This patient was a homozygote for inactivating mutation: a one-base insertion, A764, in a codon for Met^255^, thereby resulting in a frameshift. Upon transfection of *PEX19* into a CHO *pex19* mutant ZP119 devoid of peroxisomal remnants called ghosts, most striking was formation of peroxisomal membranes, prior to the import of matrix proteins (Matsuzono et al., 1999; Sacksteder et al., 2000). This was the first demonstration of the membrane assembly process during peroxisome biogenesis, particularly differentiated from the soluble protein import.

***Pex16p***. Fibroblasts from a ZS patient of CG9 (D) are deficient in peroxisomal membrane remnants, as in *PEX19*-defective fibroblasts of CG14 (J). Expression of *PEX16* encoding 336-amino-acid peroxisomal membrane protein restored peroxisomal membrane biogenesis and matrix protein import in CG9 (D) fibroblasts (Honsho et al., 1998; South and Gould, 1999), of which mutation was a homozygous nonsense mutation R176ter (Honsho et al., 1998). More recently, other mutations are identified: exon 10 skip (Shimozawa et al., 2002) and five novel homozygous mutations (Ebberink et al., 2010).

***Pex3p***. Mammalian *PEX3* encodes 42-kDa integral membrane protein of peroxisomes (Ghaedi et al., 2000a,b). Upon expression of *PEX3* in a CHO *pex3* mutant (Ghaedi et al., 2000b) and fibroblasts from three ZS patients of CG12 (G) (Ghaedi et al., 2000a), peroxisomal membrane vesicles were assembled prior to the import of soluble proteins (Ghaedi et al., 2000a; Muntau et al., 2000; Shimozawa et al., 2000; South et al., 2000; Fujiki et al., 2006a; Fujiki, 2011), as in *pex19* and *pex16* patients-derived cells (see above), likewise implying the temporally differentiated translocation of matrix proteins into peroxisomal membrane vesicles. Two types of mutations, exon 11 deletion and a single-nucleotide insertion in the codon for Val^182^ in exon 7, in *PEX3* were identified in the ZS patients (Ghaedi et al., 2000a; Muntau et al., 2000; Shimozawa et al., 2000).

Taken together, Pex3p, Pex16p, and Pex19p are categorized as a peroxin exclusively required for the assembly of peroxisome membranes. They function as essential factors in the transport process of membrane proteins and membrane vesicle assembly in a concerted manner. Two distinct pathways were recently suggested for the import of PMPs: a Pex19p- and Pex3p-dependent class I pathway for PMP-import complex, except for Pex3p (Fang et al., 2004; Matsuzono et al., 2006) and a Pex19p- and Pex16p-dependent class II pathway for Pex3p (Matsuzaki and Fujiki, 2008) (Figures 2, 3). It is noteworthy that C-tailed anchor-type peroxin Pex26p, the recruiter of Pex1p-Pex6p complex, is transported in a Pex19p-dependent (Halbach et al., 2006), class I pathway (Yagita et al., 2013), which is distinct from the GET3-dependent topogenesis of yeast Pex15p, a functional ortholog of Pex26p (Schuldiner et al., 2008).

#### Involvement of ER in peroxisome biogenesis

In regard to involvement of the ER in peroxisome biogenesis, ER was postulated to provide the initial “seed” for recruiting other components required for peroxisome assembly (Kim et al., 2006; Ma et al., 2011; Tabak et al., 2013). Several groups suggested a different view of peroxisomal membrane biogenesis that peroxisomes are formed from ER upon induction of Pex3p (Hoepfner et al., 2005; Kragt et al., 2005; Kim et al., 2006), although the significance of such observations remains under debate. Recently, a study (Motley and Hettema, 2007) suggest that peroxisomes are generally formed by growth and division under normal conditions and that only under a condition where no peroxisome is present in a cell, they can be formed from the ER after the expression of the complementing *PEX* gene, whilst another study (Van Der Zand et al., 2010) proposes that all peroxisomal membrane proteins are transported via ER. Meanwhile, we demonstrated that Pex3p, the membrane receptor for Pex19p-complexes with PMPs including Pex16p, is directly targeted to peroxisomes in a Pex19p-Pex16p dependent class II pathway in mammalian cells such as CHO and human cell lines (Matsuzaki and Fujiki, 2008). Moreover, it is noteworthy that several peroxisomal membrane proteins might be transported to peroxisomes via ER (Lam et al., 2010; Agrawal et al., 2011; Yonekawa et al., 2011), likely implying a sort of semi-autonomous property of peroxisomes. At any event, the issue with respect to how peroxisome membrane is assembled is one of the important and of highly interesting problems to be tackled (Ma et al., 2011; Fujiki et al., 2012; Tabak et al., 2013).

### Matrix protein import

Ten peroxins including Pex1p, Pex2p, Pex5p, Pex6p, Pex7p, Pex10p, Pex12p, Pex13p, Pex14p, and Pex26p are involved in protein import into peroxisomal matrix (Figure 2) (Fujiki et al., 2006a).

#### Peroxisome-cytoplasmic shuttling of import receptors

PTS1 and PTS2 proteins are recognized by Pex5p and Pex7p, respectively, in the cytoplasm. In mammalian cells, PTS1 proteins are transported by homo- and hetero-oligomers of Pex5pS and Pex5pL to peroxisomes, where Pex14p functions as the initial site of an 800-kDa “docking complex.” Pex5pL translocates the Pex7p–PTS2 protein complex to Pex14p (Otera et al., 2002; Miyata and Fujiki, 2005). After releasing the cargoes, Pex5p and Pex7p translocate to a 500-kDa “translocation complex” comprising the RING peroxins, Pex2p, Pex10p and Pex12p (Miyata and Fujiki, 2005). Both Pex5p and Pex7p finally translocate back to the cytosol (Dammai and Subramani, 2001; Gouveia et al., 2003; Nair et al., 2004; Miyata and Fujiki, 2005; Platta et al., 2005; Miyata et al., 2009). At the terminal step of the protein import reaction, AAA peroxins, Pex1p and Pex6p, recruited to Pex26p (Pex15p in yeast) on peroxisomes catalyze the ATP-dependent export of Pex5p (Miyata and Fujiki, 2005; Platta et al., 2005). Ubiquitination of Pex5p is prerequisite for the Pex5p exit (Carvalho et al., 2007; Williams et al., 2007; Okumoto et al., 2011).

Mono-ubiquitination of the conserved cysteine residue at position 11 in the N-terminal region of mammalian Pex5p plays an essential role in the recycling, especially in the export step from peroxisomes to the cytosol (Grou et al., 2009; Okumoto et al., 2011; Miyata et al., 2012), as in yeast (Platta et al., 2009). A cytosolic factors, AWP1/ZFAND6 involved in the recycling of Pex5p is recently identified in mammals (Miyata et al., 2012); USP9X and Ubp15 are suggested as a potential deubiquitinase in mammals (Grou et al., 2012) and yeast (Debelyy et al., 2011), respectively. A distinct redox state may affect the recycling of Pex5p requiring Cys-ubiquitination, thereby leading as a possible cause to the phenotype of deficiency in protein import in *PEX*-defective cells.

### Redox state of normal and peroxisome-deficient cells

In peroxisomes possessing a fatty acid β-oxidation system in wild-type CHO cells, the redox state within the peroxisomes is more reductive than that in the cytosol, despite the fact that reactive oxygen species are generated within the peroxisomes (Yano et al., 2010). Moreover, to our surprise, the redox state in the cytosol of *pex* cell mutants is more reductive than that of the wild-type CHO cells (Yano et al., 2010). Such distinct redox state may affect the recycling of Pex5p requiring Cys-ubiquitination, thereby leading as a possible cause to the phenotype of deficiency in protein import in *PEX*-defective cells including cell lines from patients with PBDs. A potential way to cure the PBD patients may be a screening for agents that moderate the abnormal cytosolic redox state in the *pex* cell lines including the cells with nonredox-sensitive mutations in *PEX*s. It is noteworthy that in *P. pastoris* PTS1-cargo release from Pex5p is achieved by a redox-regulated oligomer to dimer transition of Pex5p and aided by Pex8p (Ma et al., 2013). Interestingly, intraperoxisomal redox status is strongly influenced by environmental growth conditions (Ivashchenko et al., 2011).

## Gene defects of proteins for peroxisomal morphogenesis

Three isoforms of Pex11p family, Pex11pα (Abe et al., 1998; Li et al., 2002a), Pex11pβ (Abe and Fujiki, 1998; Schrader et al., 1998; Li et al., 2002b), and Pex11pγ (Li et al., 2002a; Tanaka et al., 2003), are identified as factors involved in morphogenesis of peroxisomes in mammals (Kobayashi et al., 2007; Delille et al., 2010; Koch et al., 2010; Itoyama et al., 2013). In mammalian cells, dynamin-like protein 1 (DLP1) (Koch et al., 2003; Li and Gould, 2003; Tanaka et al., 2006; Waterham et al., 2007), fission 1 (Fis1) (Koch et al., 2005; Kobayashi et al., 2007), and mitochondrial fission factor (Mff) (Gandre-Babbe and Van Der Bliek, 2008; Otera et al., 2010; Koch and Brocard, 2012; Itoyama et al., 2013) are shown to be involved in the fission of peroxisomes.

In regard to peroxisomal dysmorphogenesis in humans, only two patients have been identified with a different defect in any of the proteins involved in the proliferation and division of peroxisomes. The first reported patient was a severely affected female patient, who died 1 month after birth and postmortally was found to have a dominant-negative heterozygous mutation in the *DLP1* gene, which resulted in a severe fission defect of both peroxisomes and mitochondria (Waterham et al., 2007). More recently, the first patient with a defect of peroxisomal division due to a homozygous nonsense mutation in the *PEX11β* gene was reported as the 14th CG (CG16) of PBDs (Ebberink et al., 2012) (Table 1).

## Perspective

Mammalian cell mutants of 15 CGs defective of peroxisome biogenesis have been identified, including PBD patients' fibroblasts and CHO mutant cell lines (Table 1). Pathogenic genes are now elucidated for all of PBD CGs. Biochemical functions of peroxins involved in the import of matrix proteins are better elucidated, whilst molecular mechanisms underlying the membrane assembly are less understood. Defects in peroxisomal morphogenesis have also been recently reported. Investigations using the cloned peroxins and *pex* mutants including CHO mutants and those from PBD patients will shed light on the mechanisms involved in biogenesis and morphogenesis of peroxisomes and pathogenesis of PBDs.

### Conflict of interest statement

The authors declare that the research was conducted in the absence of any commercial or financial relationships that could be construed as a potential conflict of interest.

## Abbreviations

CG: complementation group
CHO: Chinese hamster ovary
DLP1: dynamin-like protein 1
EST: expressed sequence tag
PBD: peroxisome biogenesis disorder
PTS: peroxisomal targeting signal
ZS: Zellweger syndrome.

## References

[B1] Abe I. Fujiki Y. (1998). cDNA cloning and characterization of a constitutively expressed isoform of the human peroxin Pex11p. Biochem. Biophys. Res. Commun. 252, 529–533 10.1006/bbrc.1998.9684 9826565

[B2] Abe I. Okumoto K. Tamura S. Fujiki Y. (1998). Clofibrate-inducible, 28-kDa peroxisomal integral membrane protein is encoded by PEX11. FEBS Lett. 431, 468–472 10.1016/S0014-5793(98)00815-1 9714566

[B3] Agrawal G. Joshi S. Subramani S. (2011). Cell-free sorting of peroxisomal membrane proteins from the endoplasmic reticulum. Proc. Natl. Acad. Sci. U.S.A. 108, 9113–9118 10.1073/pnas.1018749108 21576455 PMC3107335

[B4] Baerends R. J. S. Rasmussen S. W. Hilbrands R. E. Van Der Heide M. Faber K. N. Reuvekamp P. T. W. (1996). The *Hansenula polymorpha PER9* gene encodes a peroxisomal membrane protein essential for peroxisome assembly and integrity. J. Biol. Chem. 271, 8887–8894 10.1074/jbc.271.15.8887 8621531

[B5] Carvalho A. F. Pinto M. P. Grou C. P. Alencastre I. S. Fransen M. Sá-Miranda C. (2007). Ubiquitination of mammalian Pex5p, the peroxisomal import receptor. J. Biol. Chem. 282, 31267–31272 10.1074/jbc.M706325200 17726030

[B6] Cregg J. M. Vankiel I. J. Sulter G. J. Veenhuis M. Harder W. (1990). Peroxisome-deficient mutants of *Hansenula polymorpha*. Yeast 6, 87–97 10.1002/yea.320060202

[B7] Dammai V. Subramani S. (2001). The human peroxisomal targeting signal receptor, Pex5p, is translocated into the peroxisomal matrix and recycled to the cytosol. Cell 105, 187–196 10.1016/S0092-8674(01)00310-5 11336669

[B8] Debelyy M. O. Platta H. W. Saffian D. Hensel A. Thoms S. Meyer H. E. (2011). Ubp15p, a ubiquitin hydrolase associated with the peroxisomal export machinery. J. Biol. Chem. 286, 28223–28234 10.1074/jbc.M111.238600 21665945 PMC3151067

[B9] Delille H. K. Agricola B. Guimaraes S. C. Borta H. Lüers G. H. Fransen M. (2010). Pex11pβ-mediated growth and division of mammalian peroxisomes follows a maturation pathway. J. Cell Sci. 123, 2750–2762 10.1242/jcs.062109 20647371

[B10] Distel B. Erdmann R. Gould S. J. Blobel G. Crane D. I. Cregg J. M. (1996). A unified nomenclature for peroxisome biogenesis factors. J. Cell Biol. 135, 1–3 10.1083/jcb.135.1.1 8858157 PMC2121017

[B11] Ebberink M. S. Csanyi B. Chong W. K. Denis S. Sharp P. Mooijer P. (2010). Identification of an unusual variant peroxisome biogenesis disorder caused by mutations in the *PEX16* gene. J. Med. Genet. 47, 608–615 10.1136/jmg.2009.074302 20647552

[B12] Ebberink M. S. Koster J. Visser G. Van Spronsen F. Stolte-Dijkstra I. Smit G. P. A. (2012). A novel defect of peroxisome division due to a homozygous non-sense mutation in the *PEX11*β gene. J. Med. Genet. 49, 307–313 10.1136/jmedgenet-2012-100778 22581968

[B13] Eitzen G. A. Szilard R. K. Rachubinski R. A. (1997). Enlarged peroxisomes are present in oleic acid-grown *Yarrowia lipolytica* overexpressing the *PEX16* gene encoding an intraperoxisomal peripheral membrane peroxin. J. Cell Biol. 137, 1265–1278 10.1083/jcb.137.6.1265 9182661 PMC2132528

[B14] Erdmann R. Veenhuis M. Mertens D. Kunau W.-H. (1989). Isolation of peroxisome-deficient mutants of *Saccharomyces cerevisiae*. Proc. Natl. Acad. Sci. U.S.A. 86, 5419–5423 10.1073/pnas.86.14.5419 2568633 PMC297634

[B15] Fang Y. Morrell J. C. Jones J. M. Gould S. J. (2004). PEX3 functions as a PEX19 docking factor in the import of class I peroxisomal membrane proteins. J. Cell Biol. 164, 863–875 10.1083/jcb.200311131 15007061 PMC2172291

[B16] Fujiki Y. (1997). Molecular defects in genetic diseases of peroxisomes. Biochim. Biophys. Acta 1361, 235–250 10.1016/S0925-4439(97)00051-3 9375798

[B17] Fujiki Y. (2000). Peroxisome biogenesis and peroxisome biogenesis disorders. FEBS Lett. 476, 42–46 10.1016/S0014-5793(00)01667-7 10878247

[B18] Fujiki Y. (2003). Peroxisome biogenesis disorders, in Nature Encyclopedia of the Human Genome, ed Cooper. D. N. (London: Nature Publishing Group), 541–547

[B19] Fujiki Y. (2011). Peroxisome biogenesis disorders, in Encyclopedia of Life Sciences (Chichester: John Wiley & Sons). 10.1002/9780470015902.a0006109.pub2

[B20] Fujiki Y. Matsuzono Y. Matsuzaki T. Fransen M. (2006a). Import of peroxisomal membrane proteins: the interplay of Pex3p- and Pex19p-mediated interactions. Biochim. Biophys. Acta 1763, 1639–1646 10.1016/j.bbamcr.2006.09.030 17069900

[B21] Fujiki Y. Okumoto K. Kinoshita N. Ghaedi K. (2006b). Lessons from peroxisome-deficient Chinese hamster ovary (CHO) cell mutants. Biochim. Biophys. Acta 1763, 1374–1381 10.1016/j.bbamcr.2006.09.012 17045664

[B22] Fujiki Y. Yagita Y. Matsuzaki T. (2012). Peroxisome biogenesis disorders: molecular basis for impaired peroxisomal membrane assembly- In metabolic functions and biogenesis of peroxisomes in health and disease. Biochim. Biophys. Acta 1822, 1337–1342 10.1016/j.bbadis.2012.06.004 22705440

[B23] Gandre-Babbe S. Van Der Bliek A. M. (2008). The novel tail-anchored membrane protein Mff controls mitochondrial and peroxisomal fission in mammalian cells. Mol. Biol. Cell. 19, 2402–2412 10.1091/mbc.E07-12-1287 18353969 PMC2397315

[B24] Ghaedi K. Honsho M. Shimozawa N. Suzuki Y. Kondo N. Fujiki Y. (2000a). *PEX3* is the causal gene responsible for peroxisome membrane assembly-defective Zellweger syndrome of complementation group G. Am. J. Hum. Genet. 67, 976–981 10.1086/303086 10968777 PMC1287899

[B25] Ghaedi K. Tamura S. Okumoto K. Matsuzono Y. Fujiki Y. (2000b). The peroxin Pex3p initiates membrane assembly in peroxisome biogenesis. Mol. Biol. Cell 11, 2085–2102 10.1091/mbc.11.6.2085 10848631 PMC14905

[B26] Götte K. Girzalsky W. Linkert M. Baumgart E. Kammerer S. Kunau W.-H. (1998). Pex19p, a farnesylated protein essential for peroxisome biogenesis. Mol. Cell. Biol. 18, 616–628 9418908 10.1128/mcb.18.1.616 PMC121529

[B27] Gould S. J. McCollum D. Spong A. P. Heyman J. A. Subramani S. (1992). Development of the yeast *Pichia pastoris* as a model organism for a genetic and molecular analysis of peroxisome assembly. Yeast 8, 613–628 10.1002/yea.320080805 1441741

[B28] Gould S. J. Valle D. (2000). Peroxisome biogenesis disorders: genetics and cell biology. Trends Genet. 16, 340–345 10.1016/S0168-9525(00)02056-4 10904262

[B29] Gouveia A. M. Guimaraes C. P. Oliveira M. E. Reguenga C. Sa-Miranda C. Azevedo J. E. (2003). Characterization of the peroxisomal cycling receptor, Pex5p, using a cell-free *in vitro* import system. J. Biol. Chem. 278, 226–232 10.1074/jbc.M209498200 12411433

[B30] Grou C. P. Carvalho A. F. Pinto M. P. Huybrechts S. J. Sá-Miranda C. (2009). Properties of the ubiquitin-Pex5p thiol ester conjugate. J. Biol. Chem. 284, 10504–10513 10.1074/jbc.M808978200 19208625 PMC2667737

[B31] Grou C. P. Francisco T. Rodrigues T. A. Freitas M. O. Pinto M. P. Carvalho A. F. (2012). Identification of ubiquitin-specific protease 9X (USP9X) as a deubiquitinase acting on ubiquitin-peroxin 5 (PEX5) thioester conjugate. J. Biol. Chem. 287, 12815–12827 10.1074/jbc.M112.340158 22371489 PMC3339989

[B32] Halbach A. Landgraf C. Lorenzen S. Rosenkranz K. Volkmer-Engert R. Erdmann R. (2006). Targeting of the tail-anchored peroxisomal membrane proteins PEX26 and PEX15 occurs through C-terminal PEX19-binding sites. J. Cell Sci. 119, 2508–2517 10.1242/jcs.02979 16763195

[B99] Hayashi M. Nishimura M. (2006). *Arabidopsis thaliana*—a model organism to study plant peroxisomes. Biochim. Biophys. Acta 1763, 1382–1391 10.1016/j.bbamcr.2006.08.014 17005266

[B33] Hettema E. H. Girzalsky W. Van Den Berg M. Erdmann R. Distel B. (2000). *Saccharomyces cerevisiae* Pex3p and Pex19p are required for proper localization and stability of peroxisomal membrane proteins. EMBO J. 19, 223–233 10.1093/emboj/19.2.223 10637226 PMC305556

[B34] Hoepfner D. Schildknegt D. Braakman I. Philippsen P. Tabak H. F. (2005). Contribution of the endoplasmic reticulum to peroxisome formation. Cell 122, 85–95 10.1016/j.cell.2005.04.025 16009135

[B35] Honsho M. Tamura S. Shimozawa N. Suzuki Y. Kondo N. Fujiki Y. (1998). Mutation in *PEX16* is causal in the peroxisome-deficient Zellweger syndrome of complementation group D. Am. J. Hum. Genet. 63, 1622–1630 10.1086/302161 9837814 PMC1377633

[B36] Itoyama A. Michiyuki S. Honsho M. Yamamoto T. Moser A. Yoshida Y. (2013). Mff functions with Pex11pβ and DLP1 in peroxisomal fission. Biol. Open 2, 998–1006 10.1242/bio.20135298 24167709 PMC3798195

[B37] Ivashchenko O. Van Veldhoven P. P. Brees C. Ho Y. S. Terlecky S. R. Fransen M. (2011). Intraperoxisomal redox balance in mammalian cells: oxidative stress and interorganellar cross-talk. Mol. Biol. Cell 22, 1440–1451 10.1091/mbc.E10-11-0919 21372177 PMC3084667

[B38] Kammerer S. Arnold N. Gutensohn W. Mewes H.-W. Kunau W.-H. Heofler G. (1997). Genomic organization and molecular characterization of a gene encoding HsPxF, a human peroxisomal farnesylated protein. Genomics 45, 200–210 10.1006/geno.1997.4914 9339377

[B39] Kammerer S. Holzinger A. Welsch U. Roscher A. A. (1998). Cloning and characterization of the gene encoding the human peroxisomal assembly protein Pex3p. FEBS Lett. 429, 53–60 10.1016/S0014-5793(98)00557-2 9657383

[B40] Kim P. K. Mullen R. T. Schumann U. Lippincott-Schwartz J. (2006). The origin and maintenance of mammalian peroxisomes involves a de novo PEX16-dependent pathway from the ER. J. Cell Biol. 173, 521–532 10.1083/jcb.200601036 16717127 PMC2063862

[B41] Kobayashi S. Tanaka A. Fujiki Y. (2007). Fis1, DLP1, and Pex11p coordinately regulate peroxisome morphogenesis. Exp. Cell Res. 313, 1675–1686 10.1016/j.yexcr.2007.02.028 17408615

[B42] Koch A. Thiemann M. Grabenbauer M. Yoon Y. McNiven M. A. Schrader M. (2003). Dynamin-like protein 1 is involved in peroxisomal fission. J. Biol. Chem. 278, 8597–8605 10.1074/jbc.M211761200 12499366

[B43] Koch A. Yoon Y. Bonekamp N. A. McNiven M. A. Schrader M. (2005). A role for Fis1 in both mitochondrial and peroxisomal fission in mammalian cells. Mol. Biol. Cell 16, 5077–5086 10.1091/mbc.E05-02-0159 16107562 PMC1266408

[B44] Koch J. Brocard C. (2012). PEX11 proteins attract Mff and human Fis1 to coordinate peroxisomal fission. J. Cell Sci. 125, 3813–3826 10.1242/jcs.102178 22595523

[B45] Koch J. Pranjic K. Huber A. Ellinger A. Hartig A. Kragler F. (2010). PEX11 family members are membrane elongation factors that coordinate peroxisome proliferation and maintenance. J. Cell Sci. 123, 3389–3400 10.1242/jcs.064907 20826455

[B46] Kragt A. Voorn-Brouwer T. Van Den Berg M. Distel B. (2005). Endoplasmic reticulum-directed Pex3p routes to peroxisomes and restores peroxisome formation in a *Saccharomyces cerevisiae pex3Δ* strain. J. Biol. Chem. 280, 34350–34357 10.1074/jbc.M505432200 16100114

[B47] Kunau W.-H. (1998). Peroxisome biogenesis from yeast to man. Curr. Opin. Microbiol. 1, 232–237 10.1016/S1369-5274(98)80016-7 10066486

[B48] Lam S. K. Yoda N. Schekman R. (2010). A vesicle carrier that mediates peroxisome protein traffic from the endoplasmic reticulum. Proc. Natl. Acad. Sci. U.S.A. 107, 21523–21528 10.1073/pnas.1013397107 21098289 PMC3003071

[B49] Lazarow P. B. (2003). Peroxisome biogenesis: advances and conundrums. Curr. Opin. Cell Biol. 15, 489–497 10.1016/S0955-0674(03)00082-6 12892791

[B50] Li X. Baumgart E. Dong G.-X. Morrell J. C. Jimenez-Sanchez G. Valle D. (2002a). PEX11α is required for peroxisome proliferation in response to 4-phenylbutyrate but is dispensable for peroxisome proliferator-activated receptor alpha-mediated peroxisome proliferation. Mol. Cell. Biol. 22, 8226–8240 10.1128/MCB.22.23.8226-8240.2002 12417726 PMC134051

[B51] Li X. Baumgart E. Morrell J. C. Jimenez-Sanchez G. Valle D. Gould S. J. (2002b). PEX11β deficiency is lethal and impairs neuronal migration but does not abrogate peroxisome function. Mol. Cell. Biol. 22, 4358–4365 10.1128/MCB.22.12.4358-4365.2002 12024045 PMC133847

[B52] Li X. Gould S. J. (2003). The dynamin-like GTPase DLP1 is essential for peroxisome division and is recruited to peroxisomes in part by PEX11. J. Biol. Chem. 278, 17012–17020 10.1074/jbc.M212031200 12618434

[B53] Liu H. Tan X. Veenhuis M. McCullum D. Cregg J. M. (1992). An efficient screen for peroxisome-deficient mutants of *Pichia pastoris*. J. Bacteriol. 174, 4943–4951 1629154 10.1128/jb.174.15.4943-4951.1992 PMC206307

[B54] Ma C. Agrawal G. Subramani S. (2011). Peroxisome assembly: matrix and membrane protein biogenesis. J. Cell Biol. 193, 7–16 10.1083/jcb.201010022 21464226 PMC3082194

[B55] Ma C. Hagstrom D. Polley S. G. Subramani S. (2013). Redox-regulated cargo binding and release by the peroxisomal targeting signal receptor, Pex5. J. Biol. Chem. 288, 27220–27231 10.1074/jbc.M113.492694 23902771 PMC3779719

[B56] Matsumoto N. Tamura S. Moser A. Moser H. W. Braverman N. Suzuki Y. (2001). The peroxin Pex6p gene is impaired in peroxisome biogenesis disorders of complementation group 6. J. Hum. Genet. 46, 273–277 10.1007/s100380170078 11355018

[B57] Matsuzaki T. Fujiki Y. (2008). The peroxisomal membrane-protein import receptor Pex3p is directly transported to peroxisomes by a novel Pex19p- and Pex16p-dependent pathway. J. Cell Biol. 183, 1275–1286 10.1083/jcb.200806062 19114594 PMC2606968

[B58] Matsuzono Y. Kinoshita N. Tamura S. Shimozawa N. Hamasaki M. Ghaedi K. (1999). Human *PEX19*: cDNA cloning by functional complementation, mutation analysis in a patient with Zellweger syndrome and potential role in peroxisomal membrane assembly. Proc. Natl. Acad. Sci. U.S.A. 96, 2116–2121 10.1073/pnas.96.5.2116 10051604 PMC26746

[B59] Matsuzono Y. Matsuzaki T. Fujiki Y. (2006). Functional domain mapping of peroxin Pex19p: interaction with Pex3p is essential for function and translocation. J. Cell Sci. 119, 3539–3550 10.1242/jcs.03100 16895967

[B60] Miyata N. Fujiki Y. (2005). Shuttling mechanism of peroxisome targeting signal type 1 receptor Pex5: ATP-independent import and ATP-dependent export. Mol. Cell. Biol. 25, 10822–10832 10.1128/MCB.25.24.10822-10832.2005 16314507 PMC1316942

[B61] Miyata N. Hosoi K. Mukai S. Fujiki Y. (2009). *In vitro* import of peroxisome-targeting signal 2 (PTS2) receptor Pex7p into peroxisomes. Biochim. Biophys. Acta 1793, 860–870 10.1016/j.bbamcr.2009.02.007 19264098

[B62] Miyata N. Okumoto K. Mukai S. Noguchi M. Fujiki Y. (2012). AWP1/ZFAND6 functions in Pex5 export by interacting with Cys-monoubiquitinated Pex5 and Pex6 AAA ATPase. Traffic 13, 168–183 10.1111/j.1600-0854.2011.01298.x 21980954

[B63] Motley A. M. Hettema E. H. (2007). Yeast peroxisomes multiply by growth and division. J. Cell Biol. 178, 399–410 10.1083/jcb.200702167 17646399 PMC2064844

[B64] Muntau A. C. Mayerhofer P. U. Paton B. C. Kammerer S. Roscher A. A. (2000). Defective peroxisome membrane synthesis due to mutations in human *PEX3* causes Zellweger syndrome, complementation group G. Am. J. Hum. Genet. 67, 967–975 10.1086/303071 10958759 PMC1287898

[B65] Nair D. M. Purdue P. E. Lazarow P. B. (2004). Pex7p translocates in and out of peroxisomes in *Saccharomyces cerevisiae*. J. Cell Biol. 167, 599–604 10.1083/jcb.200407119 15545321 PMC2172567

[B66] Nuttley W. M. Brade A. M. Gaillardin C. Eitzen G. A. Glover J. R. Aitchison J. D. (1993). Rapid identification and characterization of peroxisomal assembly mutants in *Yarrowia lipolytica*. Yeast 9, 507–517 10.1002/yea.320090506

[B67] Okumoto K. Misono S. Miyata N. Matsumoto Y. Mukai S. Fujiki Y. (2011). Cysteine ubiquitination of PTS1 receptor Pex5p regulates Pex5p recycling. Traffic 12, 1067–1083 10.1111/j.1600-0854.2011.01217.x 21554508

[B68] Otera H. Setoguchi K. Hamasaki M. Kumashiro T. Shimizu N. Fujiki Y. (2002). Peroxisomal targeting signal receptor Pex5p interacts with cargoes and import machinery components in a spatiotemporally differentiated manner: conserved Pex5p WXXXF/Y motifs are critical for matrix protein import. Mol. Cell. Biol. 22, 1639–1655 10.1128/MCB.22.6.1639-1655.2002 11865044 PMC135613

[B69] Otera H. Wang C. Cleland M. M. Setoguchi K. Yokota S. Youle R. J. (2010). Mff is an essential factor for mitochondrial recruitment of Drp1 during mitochondrial fission in mammalian cells. J. Cell Biol. 191, 1141–1158 10.1083/jcb.201007152 21149567 PMC3002033

[B70] Otzen M. Perband U. Wang D. Baerends R. J. Kunau W. H. Veenhuis M. (2004). *Hansenula polymorpha* Pex19p is essential for the formation of functional peroxisomal membranes. J. Biol. Chem. 279, 19181–19190 10.1074/jbc.M314275200 14981078

[B71] Platta H. W. Grunau S. Rosenkranz K. Girzalsky W. Erdmann R. (2005). Functional role of the AAA peroxins in dislocation of the cycling PTS1 receptor back to the cytosol. Nat. Cell Biol. 7, 817–822 10.1038/ncb1281 16007078

[B72] Platta H. W. Magraoui F. E. Bäumer B. E. Schlee D. Girzalsky W. Erdmann R. (2009). Pex2 and Pex12 function as protein-ubiquitin ligases in peroxisomal protein import. Mol. Cell. Biol. 29, 5505–5516 10.1128/MCB.00388-09 19687296 PMC2756880

[B73] Sacksteder K. A. Jones J. M. South S. T. Li X. Liu Y. Gould S. J. (2000). PEX19 binds multiple peroxisomal membrane proteins, is predominantly cytoplasmic, and is required for peroxisome membrane synthesis. J. Cell Biol. 148, 931–944 10.1083/jcb.148.5.931 10704444 PMC2174547

[B74] Schatz G. Dobberstein B. (1996). Common principles of protein translocation across membranes. Science 271, 1519–1526 10.1126/science.271.5255.1519 8599107

[B75] Schrader M. Reuber B. E. Morrell J. C. Jimenez-Sanchez G. Obie C. Stroh T. A. (1998). Expression of *PEX11*β mediates peroxisome proliferation in the absence of extracellular stimuli. J. Biol. Chem. 273, 29607–29614 10.1074/jbc.273.45.29607 9792670

[B76] Schuldiner M. Metz J. Schmid V. Denic V. Rakwalska M. Schmitt H. D. (2008). The GET complex mediates insertion of tail-anchored proteins into the ER membrane. Cell 134, 634–645 10.1016/j.cell.2008.06.025 18724936 PMC2572727

[B77] Shimozawa N. Nagase T. Takemoto Y. Suzuki Y. Fujiki Y. Wanders R. J. A. (2002). A novel aberrant splicing mutation of the PEX16 gene in two patients with Zellweger syndrome. Biochem. Biophys. Res. Commun. 292, 109–112 10.1006/bbrc.2002.6642 11890679

[B78] Shimozawa N. Suzuki Y. Zhang Z. Imamura A. Ghaedi K. Fujiki Y. (2000). Identification of *PEX3* as the gene mutated in a Zellweger syndrome patient lacking peroxisomal remnant structures. Hum. Mol. Genet. 9, 1995–1999 10.1093/hmg/9.13.1995 10942428

[B79] Shimozawa N. Tsukamoto T. Nagase T. Takemoto Y. Koyama N. Suzuki Y. (2004). Identification of a new complementation group of the peroxisome biogenesis disorders and *PEX14* as the mutated gene. Hum. Mutat. 23, 552–558 10.1002/humu.20032 15146459

[B80] Shimozawa N. Tsukamoto T. Suzuki Y. Orii T. Shirayoshi Y. Mori T. (1992). A human gene responsible for Zellweger syndrome that affects peroxisome assembly. Science 255, 1132–1134 10.1126/science.1546315 1546315

[B81] South S. T. Gould S. J. (1999). Peroxisome synthesis in the absence of preexisting peroxisomes. J. Cell Biol. 144, 255–266 10.1083/jcb.144.2.255 9922452 PMC2132891

[B82] South S. T. Sacksteder K. A. Li X. Liu Y. Gould S. J. (2000). Inhibitors of COPI and COPII do not block *PEX3*-mediated peroxisome synthesis. J. Cell Biol. 149, 1345–1360 10.1083/jcb.149.7.1345 10871277 PMC2175136

[B83] Subramani S. Koller A. Snyder W. B. (2000). Import of peroxisomal matrix and membrane proteins. Annu. Rev. Biochem. 69, 399–418 10.1146/annurev.biochem.69.1.399 10966464

[B84] Tabak H. F. Braakman I. Distel B. (1999). Peroxisomes: simple in function but complex in maintenance. Trends Cell Biol. 9, 447–453 10.1016/S0962-8924(99)01650-5 10511709

[B85] Tabak H. F. Braakman I. Van Der Zand A. (2013). Peroxisome formation and maintenance are dependent on the endoplasmic reticulum. Annu. Rev. Biochem. 82, 723–744 10.1146/annurev-biochem-081111-125123 23414306

[B86] Tanaka A. Kobayashi S. Fujiki Y. (2006). Peroxisome division is impaired in a CHO cell mutant with an inactivating point-mutation in dynamin-like protein 1 gene. Exp. Cell Res. 312, 1671–1684 10.1016/j.yexcr.2006.01.028 16529741

[B87] Tanaka A. Okumoto K. Fujiki Y. (2003). cDNA cloning and characterization of the third isoform of human peroxin Pex11p. Biochem. Biophys. Res. Commun. 300, 819–823 10.1016/S0006-291X(02)02936-4 12559946

[B88] Titorenko V. I. Rachubinski R. A. (2001). The life cycle of the peroxisome. Nat. Rev. Mol. Cell Biol. 2, 357–368 10.1038/35073063 11331910

[B89] Tsukamoto T. Miura S. Fujiki Y. (1991). Restoration by a 35K membrane protein of peroxisome assembly in a peroxisome-deficient mammalian cell mutant. Nature 350, 77–81 10.1038/350077a0 1750930

[B90] Van Der Klei I. J. Veenhuis M. (1996). Peroxisome biogenesis in the yeast *Hansenula polymorpha*: a structural and functional analysis. Ann. N. Y. Acad. Sci. 804, 47–59 10.1111/j.1749-6632.1996.tb18607.x 8993535

[B91] Van Der Zand A. Braakman I. Tabak H. F. (2010). Peroxisomal membrane proteins insert into the endoplasmic reticulum. Mol. Biol. Cell 21, 2057–2065 10.1091/mbc.E10-02-0082 20427571 PMC2883949

[B92] Waterham H. R. Koster J. Van Roermund C. W. T. Mooyer P. A. W. Wanders R. J. A. Leonard J. V. (2007). A lethal defect of mitochondrial and peroxisomal fission. N. Engl. J. Med. 356, 1736–1741 10.1056/NEJMoa064436 17460227

[B93] Weller S. Gould S. J. Valle D. (2003). Peroxisome biogenesis disorders. Annu. Rev. Genomics Hum. Genet. 4, 165–211 10.1146/annurev.genom.4.070802.110424 14527301

[B94] Wickner W. Schekman R. (2005). Protein translocation across biological membranes. Science 310, 1452–1456 10.1126/science.1113752 16322447

[B95] Williams C. Van Den Berg M. Sprenger R. R. Distel B. (2007). A conserved cysteine is essential for Pex4p-dependent ubiquitination of the peroxisomal import receptor Pex5p. J. Biol. Chem. 282, 22534–22543 10.1074/jbc.M702038200 17550898

[B96] Yagita Y. Hiromasa T. Fujiki Y. (2013). Tail-anchored PEX26 targets peroxisomes via a PEX19-dependent and TRC40-independent class I pathway. J. Cell Biol. 200, 651–666 10.1083/jcb.201211077 23460677 PMC3587837

[B97] Yano T. Oku M. Akeyama N. Itoyama A. Yurimoto H. Kuge S. (2010). A novel fluorescent sensor protein for visualization of redox states in the cytoplasm and in peroxisomes. Mol. Cell. Biol. 30, 3758–3766 10.1128/MCB.00121-10 20498274 PMC2916398

[B98] Yonekawa S. Furuno A. Baba T. Fujiki Y. Ogasawara Y. Yamamoto A. (2011). Sec16B is involved in the endoplasmic reticulum export of the peroxisomal membrane biogenesis factor peroxin 16 (Pex16) in mammalian cells. Proc. Natl. Acad. Sci. U.S.A. 108, 12746–12751 10.1073/pnas.1103283108 21768384 PMC3150892

